# Decreased ultra-processed food consumption as a mediator for lowering cardiovascular risk after a lifestyle program in pediatric obesity: a randomized clinical trial

**DOI:** 10.3389/fnut.2026.1753345

**Published:** 2026-02-11

**Authors:** Ana Ojeda-Rodríguez, José Francisco López-Gil, Ana Catalán-Lambán, M. Cristina Azcona, Amelia Marti del Moral

**Affiliations:** 1 Department of Nutrition, Food Science and Physiology, University of Navarra, Pamplona, Navarra, Spain; 2 Center of Biomedical Research in Physiopathology of Obesity and Nutrition (CIBEROBN), Institute of Health Carlos III, Madrid, Spain; 3 Lipids and Atherosclerosis Unit, Unidad de Gestión Clínica Medicina Interna, Maimonides Institute for Biomedical Research in Córdoba (IMIBIC), Reina Sofia University Hospital, University of Córdoba, Córdoba, Spain; 4 School of Medicine, Universidad Espíritu Santo, Samborondón, Ecuador; 5 Faculty of Health Sciences, Universidad Autónoma de Chile, Santiago, Chile; 6 Paediatric Endocrinology Unit, Department of Pediatrics, Clínica Universidad de Navarra, Pamplona, Spain; 7 IdiSNA (Navarra Institute for Health Research), Pamplona, Spain; 8 Institute of Nutrition and Health, University of Navarra, Pamplona, Spain

**Keywords:** diet quality, life’s simple 8 score, mediation analysis, Mediterranean diet, weight loss, pediatric obesity, ultraprocessed food

## Abstract

**Introduction:**

We conducted this study to assess changes in cardiovascular health, ultra-processed food (UPF) consumption, and their relationship after a weight loss program intervention in a pediatric population with abdominal obesity.

**Methods:**

A total of 107 participants (7–16 years old) were randomly assigned to either the intervention (moderate hypocaloric Mediterranean diet; *n* = 81) or usual care group (standard pediatric advice; *n* = 26) for an intensive 8-week lifestyle intervention (ClinicalTrials.gov, Identifier: NCT03147261). UPFs were categorized using the NOVA classification system, and cardiovascular health was defined using the American Heart Association’s Life’s Essential 8 scores (LE8, 0–100 points).

**Results:**

A significant increase in 5.94 points of LE8 (*p* < 0.001) was observed in the intervention group after the intensive phase, but not in the usual care group. The difference between groups was significant, amounting 8.41 points (*p* < 0.001). Additionally, both groups reduced their UPF consumption in 2.74 (*p* < 0.001) and 2.15 portions/day (*p* < 0.001), with the intervention group showing a greater reduction of −0.59 portions/day (*p* = 0.032). A significant negative association between changes in UPF consumption and changes in LE8 was found (*p* = 0.024) after adjusting for changes in energy intake and somatic maturity. Mediation analysis revealed that the effect of the lifestyle program on LE8 score was mediated by the decrease in UPF consumption.

**Conclusion:**

The weight loss program in a pediatric population with abdominal obesity not only improved anthropometric and clinical parameters but also reduced UPF consumption, thereby enhancing cardiovascular health.

## 1 Introduction

Childhood and adolescent obesity is a significant public health challenge in the 21st century. Globally, pediatric obesity rates are doubling every decade, with projections estimating that over 254 million children will be affected by 2030 (1). In the WHO European Region, nearly one in three children (29% of boys and 27% of girls) (2) have overweight or obese.

Obesity is a chronic condition, with a high likelihood of persisting into later life stages. Approximately 55% of children with obesity continue to experience it during adolescence, and around 80% of these individuals remain obese in adulthood (3). The increasing prevalence of obesity is associated with the emergence of comorbidities, including cardiovascular disease (CVD), which is predominantly seen in adults (4). Research indicates that reducing the severity of childhood obesity can delay or prevent the onset of CVD. Notably, adolescents with obesity and multiple cardiovascular risk factors are nearly 15 times more likely to develop CVD before the age of 50 (5). Thus, early detection of cardiovascular risk is crucial for preventing future cardiovascular incidents and reducing the associated burden (6).

In 2010, the American Heart Association (AHA) introduced “Life’s Essential 7” (LE7), a framework for defining and quantifying an ideal cardiovascular health (CVH) based on seven health factors: diet, physical activity, cigarette smoking, body mass index (BMI), total cholesterol, blood pressure (BP), and blood glucose (7). The metric was updated in 2022 to “Life’s Essential 8” (LE8) with the inclusion of sleep health (8).

Projected trends suggest that the prevalence for pediatric obesity will increase from 20% in 2020 to 33% in 2050, with inadequate physical activity and poor diet affecting nearing 60% of children, impacting over 45 million children (9). Seven studies have validated the LE8 as a robust measure of children’s CVH (10–16), with diet consistently showing the lowest prevalence of achieving an ideal score (10–15). This factor directly and indirectly influences other CVH metrics (10).

In recent decades, countries of all income levels have shifted from traditional diet to Western dietary patterns, characterized by high consumption of ultra-processed foods (UPFs) (17). These ready-to-eat foods, derived from substances extracted or refined from whole foods, rich in cosmetic additives, with few or no whole foods, have been linked to numerous health issues in children, including poor lipid profile, high BP, a high prevalence of Metabolic Syndrome and obesity (17). Additionally, they are associated with lower overall CVH (18). Therefore, it is crucial to implement interventions promoting healthy lifestyles, including healthy dietary patterns and increased physical activity, to reduce and prevent both obesity and the associated premature cardiovascular risks.

In this context, we hypothesize that a lifestyle intervention aimed at weight loss in a pediatric population with abdominal obesity could reduce UPF consumption and attenuate the risk of cardiovascular disease, as measured by LE8. The potential mediating role of UPF consumption on CVD risk will be tested to explore this relationship further.

## 2 Materials and methods

The IGENOI (Intervention of *Grupo Estudio Navarro de Obesidad Infantil*) study is a family-based lifestyle intervention study conducted as a randomized controlled clinical trial (ClinicalTrials.gov, Identifier: NCT03147261). Led by the GENOI group (Navarro Study Group of Childhood Obesity), the study took place from January 2015 to January 2019 in Pamplona, Spain. Participants were recruited from the Pediatric Endocrinology Unit of the Clinic University of Navarra, the Pediatric Department of the University Hospital Complex of Navarra and various health Centers in Pamplona. The study population consisted of children and adolescents aged 7 and 16 years with a waist circumference above the 90th percentile, according to national reference data (19). Exclusion criteria included prevalent pre-diabetes, presence of other diseases, pharmacotherapy, special diet treatment, food intolerance, regular alcohol consumption, eating disorders or psychiatric disease. The study followed the ethical standards outlined in the 2013 Declaration of Helsinki and was approved and supervised by the Human Research Ethics Committee of the University of Navarra (044/2014). Written informed consent was obtained from eligible children and their parents or legal guardians.

### 2.1 Participants

Out of an initial screening of 126 children, 121 met the inclusion criteria. Of these, 114 completed the 8-week phase, with a drop-out rate of 5.8%, primarily due to discouragement, changes of address, social problems or scheduling conflicts. One hundred and nine participants completed the dietary intake (at baseline and 8 weeks), but two subjects were identified as outlier and were removed from the statistical analysis. The final sample was 107 children and adolescents: 81 subjects of intervention group and 26 of usual care group (Supplementary Figure S1).

### 2.2 Randomization

Participants were randomly assigned to the usual care group or the intensive care group in a 1:3 allocation ratio using a computer-generated randomization sequence. The randomization sequence was generated by an independent researcher not involved in participant recruitment, enrollment, or outcome assessment, and allocation was concealed through a centralized assignment system. Randomization was performed after participant enrollment and completion of baseline assessments. The unequal allocation ratio was chosen to allow a greater number of participants to receive intensive intervention. Due to the nature of the intervention, blinding of participants and clinicians was not feasible. Randomization resulted in comparable baseline characteristics between the study groups, as shown in Table 1.

**Table 1:** Changes in anthropometric, clinical and lifestyle parameters after a lifestyle intervention in a pediatric population with obesity.

Group IG *n* = 81 UCG *n* = 26		Baseline	8 weeks	Diff.	*P*	Diff.	*P*
		Mean ± SD	Mean ± SD				
Age (years)	IG	11.5 ± 2.5					
	UCG	10.7 ± 2.4					
Sex (% F)	IG	61					
	UCG	69					
Tanner (% 1/2/3/4/5)	IG	33/22/13/7/25					
	UCG	41/7/28/3/21					
BMI-SDS	IG	2.86 ± 0.99	2.35 ± 1.03	−0.50	<0.001	−0.01	0.965
	UCG	2.90 ± 1.15	2.40 ± 1.14	−0.51	0.001		
WC (cm)	IG	86.41 ± 11.40	82.57 ± 11.51	−3.83	<0.001	0.70	0.562
	UCG	85.78 ± 11.47	81.24 ± 10.45	−4.28	<0.001		
HC (cm)	IG	99.22 ± 12.77	96.60 ± 13.39	−2.63	<0.001		
	UCG	96.78 ± 12.34	95.08 ± 11.16	−1.70	0.008	0.93	0.167
Fat (%)	IG	36.61 ± 5.52	34.04 ± 6.08	−2.57	<0.001	−1.04	0.033
	UCG	36.73 ± 7.97	35.19 ± 8.07	−1.53	<0.001		
TAG (mg/dL)	IG	83.32 ± 34.25	74.35 ± 27.38	−8.97	0.028	8.47	0.292
	UCG	97.21 ± 39.74	79.78 ± 39.91	−17.45	0.028		
Cholesterol (mg/dL)	IG	162.73 ± 25.40	150.80 ± 25.26	−11.94	<0.001	0.55	0.911
	UCG	158.60 ± 21.28	147.21 ± 24.90	−11.39	0.003		
LDL (mg/dL)	IG	98.30 ± 20.98	91.95 ± 21.54	−6.36	0.004	−1.96	0.609
	UCG	94.13 ± 17.22	89.73 ± 20.25	−4.39	0.087		
Insulin (μU/mL)	IG	15.30 ± 7.08	13.17 ± 5.96	−2.13	0.008	1.38	0.465
	UCG	20.48 ± 21.33	16.96 ± 15.03	−3.52	0.138		
QUICKI index	IG	0.33 ± 0.02	0.33 ± 0.03	0.01	0.002	0.00	0.699
	UCG	0.32 ± 0.03	0.33 ± 0.03	0.01	0.072		
HOMA index	IG	3.37 ± 1.71	2.83 ± 1.32	−0.54	0.007	0.42	0.395
	UCG	4.66 ± 5.08	3.69 ± 3.26	−0.97	0.146		
Total energy (kcal/day)	IG	2681.65 ± 585.59	1950.59 ± 366.07	−731.05	<0.001	35.03	0.793
	UCG	2729.39 ± 572.61	1963.31 ± 429.27	−766.09	<0.001		
HLD-I (0 to 36)	IG	18.00 ± 3.08	22.14 ± 2.41	4.14	<0.001	2.65	0.002
	UCG	18.28 ± 2.73	19.72 ± 2.79	1.44	0.058		
DQI-A (−33% to 100%)	IG	26.05 ± 7.82	38.16 ± 6.05	12.11	<0.001	5.33	0.020
	UCG	26.28 ± 9.00	33.05 ± 7.35	6.77	0.005		

IG, intervention group; UCG, usual care group; BMI-SDS, standard deviation score for body mass index; WC, waist circumference; TAG, triglycerides; LDL, low density cholesterol; HDL, high density cholesterol; non-HDL, total cholesterol minus HDL cholesterol; QUICKI index, quantitative insulin sensitivity check index; HOMA index, homeostasis model assessment. No significant differences between groups at baseline were found.

### 2.3 Experimental design

A multidisciplinary team comprising registered dieticians, pediatricians, physical activity experts and nurses carried out the 2 year-lifestyle intervention, which consisted of an 8-week intensive phase, followed by a 22-month follow-up period (20). This report presents data from the intensive phase. During this period, the usual care subjects received pediatric recommendations for a healthy diet based on the national guidelines of the Spanish Society of Community Nutrition (21) in one 30-min individual session with the dietitian, and 5 monitoring visits to evaluate anthropometric measurements with the research team. These recommendations focused on promoting a balanced and varied diet, healthy lifestyle habits, and adequate energy balance, with emphasis on regular physical activity and healthy culinary practices. In contrast, intervention subjects were advised to follow a moderately hypocaloric Mediterranean diet (22, 23). The dietary plan is detailed in Supplementary Table S1. Briefly, dietary intervention consists on a fixed full-day meal plan of five meals. Total daily energy was distributed among the day following the pattern: 20% on breakfast, 5%–10% on morning snack, 30%–35% on lunch, 10%–15% on afternoon snack and 20%–25% on dinner. The dietary plan was based in Mediterranean pattern; thus, it includes a high consumption of fruits, vegetables, whole grains, legumes, olive oil and minimally processed foods; a moderate consumption of dairy products, fish and poultry; and a low consumption of red meat. Intervention subjects received six 30-min individual sessions to both assess and address possible problems with dietary compliance along with anthropometric evaluation. In addition, the subjects and their parents (or legal guardians) attended a parallel group session in the third week. During the group sessions parents were told their role in the intervention and the obesity related comorbidities, while children were taught about different topics such as energy balance, portion sizes, groups of foods, the importance of the breakfast and physical activity. Both groups were encouraged to engage in an additional 200 min of physical activity per week at 60%–75% of their maximum heart rate. All participants were accompanied by their parents or legal guardians (20).

### 2.4 Anthropometric, clinical and biochemical measurements

All measurements were taken at baseline and after the 8-week intervention by trained personnel using standard procedures and using calibrated equipment. Height was obtained with a Harpenden’s stadiometer of 1 mm precision (Seca 220, Vogel and Halke, Hamburg, Germany) and body weight using a digital scale (BC-418, TANITA, Tokyo, and Japan). Body Mass Index (BMI) was calculated from the ratio of weight to height squared (kg/m^2^) and converted after into standard deviation scores (SDs) for sex and age derived from Spanish reference data according to specific cutoff points for BMI (19). Waist and hip circumferences were measured with a non-stretchable measuring tape (Type SECA 200). Somatic maturity was evaluated according to Tanner stages by the pediatricians (24). Venous blood samples were collected after an overnight fast to measure glucose, insulin, and lipid profile using standard auto analyzer techniques. Insulin resistance and sensitivity were evaluated using Homeostasis Model Assessment of Insulin Resistance (HOMA-IR) and Quantitative Insulin Sensitivity Check Index (QUICKI), respectively. HOMA-IR was calculated as [fasting glucose (mg/dL) x fasting insulin (μU/mL)]/405 (25). QUICKI was calculated as 1/[log (fasting insulin (μU/mL)) + log (fasting glucose (mg/dL))] (26). Blood Pressure (BP) was measured three times using an electronic sphygmomanometer (OMRON M6, Hoofddorp, The Netherlands) on the right arm after the children had rested quietly for 15 min.

### 2.5 Physical activity, sedentary behavior, and sleep duration

Physical Activity, sedentary behavior, and sleep duration were objectively measured during leisure time over 4 consecutive full days, 2 weekdays and 2 weekend days by triaxial accelerometry (Actigraph wGT3X-BT, Actigraph LLC, Penascola, FL, USA). Participants and parents were instructed to wear the accelerometer around the non-dominant waist all the time, including sleep time, and removing it just for water-related activities (bathing or showering). The accelerometer collected data in 60-s intervals (27), which were analyzed using ActiLife 6.0 software (Actigraph LLC, Penascola, FL, USA), and summarized as counts per min (CPM) using validated cutoff points to define time spent sedentary (<100 CPM) and moderate-to-vigorous physical activity (MVPA) (>2,296 CPM) (28). Sleep was assessed using the accelerometer Actigraph wGT3X-BT, and the data was analyzed using the Sadeh algorithm derived from fundamental research performed by Sadeh et al. (29). This algorithm was commonly used in younger adolescents (10 to 11 years old) (30).

### 2.6 Dietary intake assessment: diet quality

Trained dieticians collected dietary intake data at baseline and after an 8-week period using a semi-quantitative 136-item Food-Frequency Questionnaire (FFQ) (23), previously validated in Spain (31, 32). Diet quality was evaluated through three dietary indices that provide different insights into various aspect of one’s dietary patterns, encompassing diversity of food intake (Diet Quality Index for Adolescents, DQI-A), lifestyle habits such as physical activity (Healthy Lifestyle Diet Index, HLD-I) and adherence to the Mediterranean diet (Mediterranean Diet Quality Index, KIDMED), as previously reported (20).

The DQI-A, which was previously validated and adapted for use in adolescents (33), is composed by the sum of three categories presented in percentages: quality, diversity, and equilibrium. Its total value ranges from −33% to 100% (33). The HLD-Index is composed of 10 items, eight of which refer to the frequency of consumption of fruit, vegetables, fish and seafood, sweets, regular soft drinks, grain, dairy products, meat, and meat products. The other two components indicate the level of physical activity through measuring the time spent on moderate to vigorous physical activity versus looking at screens (34). The HLD-Index employed in this study is a modified version of the original index due to a lack of information on screen hours, with a range from 0 and 36. Finally, the KIDMED index score evaluated the adequacy of Mediterranean dietary patterns in children and adolescents, with scores ranging from 0 to 12 (35).

### 2.7 Dietary intake assessment: UPFs consumption

The estimated daily food consumption was calculated by multiplying the portion size in grams by the consumption frequency for each item. To evaluate the consumption of UPFs, we classified all FFQ items according to NOVA classification system which divides food in four groups according to its nature, extent, and purpose of industrial food processing (36). UPFs are industrial formulations of food-derived substances (oils, fats, sugars, starch, protein isolates) that contain little or no whole food and often include flavorings, colorings, emulsifiers, and other cosmetic additives (e.g., carbonated drinks; sweet and savory packaged snacks; ice-cream, chocolate, and candies (confectionery); mass-produced packaged bread and buns; margarines and spreads; cookies, pastries, cakes, and cake mixes; breakfast “cereals,” “cereal” and “energy” bars; “energy drinks”; milk drinks, “fruit” yogurts, and “fruit” drinks; cocoa drinks; meat and chicken extracts and “instant” sauces; infant formulas, follow-on milks, and other baby products; “health” and “slimming” products such as powdered or “fortified” meal and dish substitutes; and many ready-to-heat products including prepared pies and pasta and pizza dishes; poultry and fish “nuggets” and “sticks,” sausages, burgers, hot dogs, and other reconstituted meat products; and powdered and packaged “instant” soups, noodles, and desserts). The sum of UPF items was estimated using the frequency of UPF consumption per person (servings/day).

### 2.8 Life’s Essential 8 (score)

LE8 is an updated metric for assessing CVH, encompassing 4 health behaviors: diet (updated), physical activity, nicotine exposure (updated), sleep health (new); and health factors like BMI, blood lipids (updated), blood glucose (updated), and BP; each metric range from 0 to 100 points (8). Given the absence of nicotine exposure data, we included 7 metrics (physical activity, sleep health, BMI, blood lipids, blood glucose, and BP) to construct LE8 as defined by the AHA. This approach is validated by a study using data from three large nationwide prospective cohorts (NHS, NHSII, and HPFS) and a nationally representative survey (NHANES). The study demonstrated that CVH-related factors routinely measured in many research and clinical settings can accurately estimate individuals’ overall CVH, even when not all eight LE8 metrics are available (37).

### 2.9 Statistical analysis

The sample size calculation indicated that 13 subjects were needed in usual care and 39 in the intervention group, based on a 5% error rate, 90% power, a 1:3 ratio, and a mean difference of 0.50 (SD 0.47) units in BMI-SDS after the intervention (38). Variables were described using mean ± SD; all statistical analyzes were two-tailed and a *p*-value < 0.05 was considered as statistically significant. The change in each variable was calculated as the difference between post- and pre- intervention values for each subject. Student’s unpaired or paired t-test were used for comparison between groups or within group changes, respectively. Differences in fasting glucose and systolic BP (SBP) between groups, which showed baseline differences, were analyzed using ANCOVA adjusted for baseline values. Differences in LE8 and UPF consumption were evaluated using ANCOVA adjusted for changes in total energy intake and somatic maturity (Tanner stage), given the wide age range of participants and the weight-loss intervention design; this adjustment allowed isolation of the associations of interest independently of overall caloric reduction. Associations between CVH and lifestyle or clinical variables were assessed using multiple linear regression adjusted for the same covariates. Stata/MP 14.1 for Mac (version 14.1, College Station, TX: StataCorp LP, USA) was used as statistical software.

Linear regression models were fitted using bootstrapped (10,000 samples) mediation techniques contained in the PROCESS R macro (version 4.3), using Model 4. The analyzes aimed to determine whether the effect of the weight loss program (independent variable) on the change in LE8 (dependent variable) was mediated by the change in UFPs consumption (mediator). The mediation model included the following paths: (a) the effect of the weight loss program on the change in UPFs consumption (path a); (b) the effect of the change in UPFs consumption on the change in LE8 score, controlling for the weight loss program (path b); (c) the total effect of the weight loss program on the change in LE8 score (path c); (c’): the total effect of the weight loss program on the change in LE8 score after accounting for the mediator (path c’). The indirect effect (ab) was estimated using percentile bootstrap 95% confidence intervals. Given the use of modified versions of the HLD-I and LE8 scores, all analyzes involving these indices were conducted with an exploratory aim. No formal psychometric or measurement-model validation procedures were performed, as the study was not designed for instrument validation. Accordingly, the statistical analyzes focused on examining associations and mediation effects with clinical and anthropometric outcomes, rather than on evaluating the measurement properties of the indices.

## 3 Results

### 3.1 Characteristics of participants

A total of 121 children with abdominal obesity (11.25 years old; 62% girls) were randomized into two groups (usual care or intervention) for the IGENOI lifestyle intervention. Of these, 114 participants completed the 8-week intensive intervention. This sub-study consisted of 107 children (11.3 years old; 63% girls) with abdominal obesity (Supplementary Figure S1).

Baseline anthropometric, clinical and lifestyle parameters were not differ statistically between the intervention (*n* = 81) and usual care (*n* = 26) groups (Table 1). Both groups exhibited similar health behaviors, health factor scores based on LE8, overall LE8 score, CVH status and UPFs consumption at baseline (Table 2), except for significantly lower glucose levels (−3.18 mg/dL, *p* = 0.034) and a higher SBP (5.71 mm HG, *p* = 0.047) in the intervention group compared to the usual care group.

**Table 2:** Baseline variables, mean scores and % of cardiovascular health status categories based on Life’s Essential 8, and ultra-processed foods consumption.

	Intervention (*n* = 81)	Usual care (*n* = 26)	Diff.	*p-value*
	Mean ± SD	Mean ± SD		
Health behaviors scores				
Diet				
Kidmed point	5.71 ± 2.08	5.23 ± 1.82	0.48	0.297
Mean score	48.54 ± 37.46	37.50 ± 33.80	11.04	0.185
Physical activity				
Min/day	43.65 ± 23.94	47.52 ± 25.09	−3.86	0.483
Mean score	64.87 ± 29.39	70.38 ± 30.13	−5.51	0.412
Sleep health				
Hour/night	8.71 ± 0.79	8.66 ± 0.75	0.04	0.804
Mean score	81.84 ± 18.38	77.69 ± 21.41	4.15	0.343
Health factor scores				
BMI				
kg/m^2^	28.52 ± 4.57	28.05 ± 4.45	0.47	0.647
Mean score	27.59 ± 17.50	25.57 ± 16.69	2.02	0.607
Blood lipids				
HDL-c (mg/dL)	48.19 ± 10.55	44.92 ± 9.54	3.27	0.180
Non-HDL-c (mg/dL)	117.25 ± 25.63	111.92 ± 20.80	5.34	0.357
Mean score	57.02 ± 25.57	64.17 ± 24.30	−7.14	0.232
Blood glucose				
Glucose levels (mg/dL)	88.04 ± 6.25	91.22 ± 6.05	−3.18	0.034
Mean score	98.42 ± 7.84	94.78 ± 13.77	3.64	0.111
Blood pressure				
SBP (mm HG)	119.01 ± 11.98	113.31 ± 11.49	5.71	0.036
DBP (mm HG)	72.82 ± 8.23	71.46 ± 8.09	1.36	0.464
Mean score	70.57 ± 26.78	80.77 ± 24.81	−10.20	0.090
Overall CVH score				
Mean score	63.51 ± 11.44	63.76 ± 9.38	0.25	0.920
CVH status (%)				
Low (<50)	7	8	1	0.513
Moderate (50 to <80)	88	92	−4	
High (80 to <100)	5	0	5	
Optimal (100)	0	0	0	
UPFs consumption				
Portions/d	4.35 ± 2.26	4.71 ± 2.50	0.36	0.489

Data are presented as mean ± SD. BMI, body mass index; BMI-SDS, standard deviation score for body mass index; HDL-c, high density lipoprotein cholesterol; Non-HDL-c, total cholesterol minus HDL-c; SBP, systolic blood pressure; DBP, diastolic blood pressure; CVH, cardiovascular heath based on Life’s Essential 8; UPFs, ultra processed foods.

### 3.2 Life’s Essential 8

Changes in scores measured by LE8 are summarized in Table 3. The intervention and usual care groups showed a significant increase in three (*p* < 0.001) and two points (*p* = 0.001) in the KIDMED score, respectively. Both groups showed significant decreases in BMI, HDL, non-HDL and glucose levels, with the mean score for blood lipids in the LE8 significantly improved (+11.88 points, *p* < 0.001) only in the intervention group. Additionally, the intervention group increased moderate to vigorous physical activity (+5.58 min/day, *p* = 0.028), and the mean score for BMI in the LE8 (10.25 points, *p* < 0.001) with a significant difference between groups (compared to the usual care, +7.53 points, *p* = 0.021). Furthermore, a significant decrease in BP was only observed in the intervention group, with a significant difference between groups of −7.39 mmHg for SBP (*p* = 0.032), −5.18 mmHg for diastolic BP (DBP, *p* = 0.029) and in 18.57 points for BP of LE8 (*p* = 0.002) compared to the usual care group.

**Table 3:** Change in characteristics, mean scores and % of cardiovascular health (CVH) status categories based on Life’s Essential 8 after a lifestyle intervention in a pediatric population with abdominal obesity.

	Intervention (*n* = 81)	Usual care (*n* = 26)	Diff.	*p-value*
	Mean ± SD	Mean ± SD		
Health behaviors scores				
Diet				
Δ kidmed point	2.98 ± 2.34***	1.96 ± 2.55**	1.02	0.063
Δ mean score	−1.39 ± 44.11	−18.65 ± 48.69	17.26	0.096
Physical activity				
Δ min/day	5.58 ± 21.52*	0.58 ± 26.77	4.87	0.357
Δ mean score	6.54 ± 27.72*	−2 ± 33.89	8.54	0.199
Sleep health				
Δ hour/night	0.05 ± 0.89	−0.01 ± 1.50	0.06	0.800
Δ mean score	0.80 ± 23.35	−6 ± 25.50	6.80	0.221
Health factor scores				
BMI				
Δ kg/m^2^	−1.48 ± 0.98***	−1.36 ± 1.08***	−0.11	0.612
Δ mean score	10.25 ± 15.69***	2.69 ± 8.63	7.53	0.021
Blood lipids				
Δ HDL-c (mg/dL)	−3.77 ± 6.27***	−3.47 ± 7.11*	−0.29	0.856
Δ non-HDL-c (mg/dL)	−8.39 ± 18.14**	−7.91 ± 15.54*	0.48	0.901
Δ mean score	11.88 ± 22.46***	6.97 ± 20.55	4.92	0.360
Blood glucose				
Δ glucose levels (mg/dL)	−2.04 ± 7.10*	−5.82 ± 7.02**	3.77	0.362^#^
Δ mean score	1.18 ± 9.70	5.46 ± 14.05	−4.28	0.113
Blood pressure				
Δ SBP (mmHg)	−6.63 ± 10.52***	0.76 ± 13.06	−7.39	0.032^#^
Δ DBP (mmHg)	−3.51 ± 8.47**	1.68 ± 14.19	−5.18	0.029
Δ mean score	14.56 ± 25.51***	−4.00 ± 24.66	18.57	0.002

Data are presented as mean ± SD. BMI, body mass index; BMI-SDS, standard deviation score for body mass index; HDL-c, high density lipoprotein cholesterol; Non-HDL-c, total cholesterol minus HDL-c; SBP, systolic blood pressure; and DBP, diastolic blood pressure. ^*^p-value<0.05; ^**^p-value<0.010; ^***^p-value<0.001. ^#^Due to the mean differences at baseline, we used ANCOVA model adjusted for the corresponding variable at baseline.

**Figure 1:**
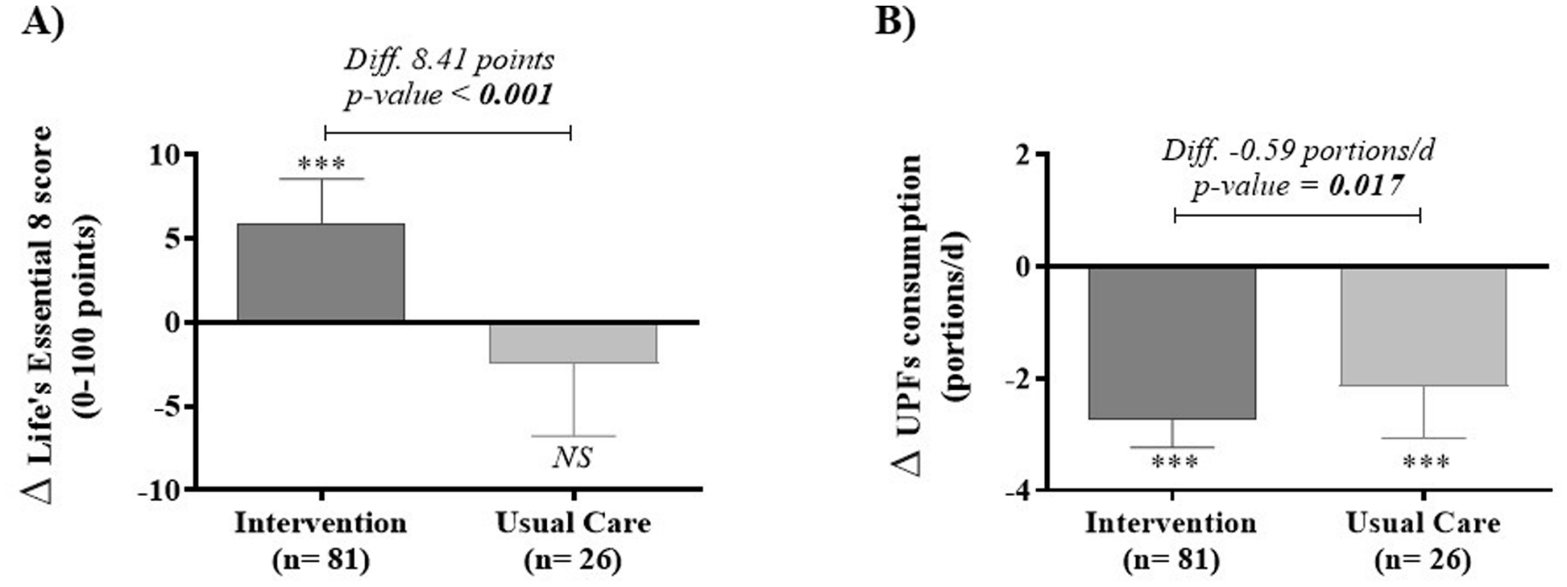
Effect of 8-week lifestyle intervention on the endpoint variables: the Life’s Essential 8 score **(A)** and UPFs consumption **(B)** in a pediatric population with abdominal obesity. Differences (Diff.) within group by student’s *t*-test analysis; differences between groups by ANCOVA adjusted for change in energy intake and somatic maturity.

In Figure 1, we show the effect of the 8-week intervention on the key endpoints. The impact of the lifestyle intervention on CVH, as measured by LE8, is shown in Figure 1A. A significant increase in +5.94 points of LE8 (*p* < 0.001) was observed in the intervention group, but not in the usual care group (−2.47 points, *p* = 0.248), with a significant difference between groups of +8.41 points (*p* < 0.001).

### 3.3 Ultra-processed food consumption

Consumption of UPF after the 8-week intervention in children with abdominal obesity is shown in Figure 1B. The intervention group showed a significant decrease about 2.74 portions of UPF per day (*p* < 0.001), while the usual care group decreased by 2.15 portions/day (*p* < 0.001). The difference between groups statistically significant (−0.59 portions per day, *p* = 0.032).

### 3.4 Others anthropometric, clinical, and lifestyle parameters

Changes in other anthropometric, clinical and lifestyle parameters not included in the LE8 are summarized in Table 1. Participants of both groups showed a significant decrease in anthropometric measurements (BMI-SDS, WC, HC, and fat mass percentage), lipids metabolism (TAG and total cholesterol) and total energy, along with an increase in DQI-A after the intervention. The intervention group also showed a significant decrease in LDL levels, insulin levels and HOMA index, while increased QUICKI index and HLD-I. Compared to the usual care group, participants in the intervention group showed a greater reduction in fat mass (−1.04%, *p* = 0.033) and a greater increase in HLD-I (2.65 points, *p* = 0.002) and DQI-A (5.33%, *p* = 0.020).

### 3.5 Associations between Life’s Essential 8 and study variables

Multiple linear regressions were performed to assess the association between changes in LE8 and changes in anthropometric, clinical, and lifestyle measures (Table 4). We found a negative fitted association between changes in LE8 and changes in BMI-SDS (*β* −6.832, *p* = 0.039), HC (*β* −0.805, *p* = 0.010), total cholesterol (*β* −0.194, *p* = 0.004), LDL (*β* −0.211, *p* = 0.015), non-HDL (*β* −0.221, *p* = 0.015), insulin (*β* −0.370, *p* = 0.048), SBP (*β* −0.366, *p* < 0.001) and DBP (*β* −0.104, *p* = 0.026); and a positive association between changes in LE8 and changes in QUICKI index (*β* 145.393, *p* = 0.020), DQI-A (*β* 0.353, *p* = 0.006), HLD-I (*β* 1.573, *p* < 0.001) and KIDMED (*β* 2.859, *p* < 0.001).

**Table 4:** Association between changes in anthropometric, clinical and lifestyle measurements with changes in Life’s Essential 8 score after a lifestyle intervention in a pediatric population with abdominal obesity.

	Δ Life’s Essential 8 (*n* = 107)					
	Crude regression			Adjusted model		
	R^2^	β	*p-value*	R^2^	β	*p-value*
Anthropometric variables						
Δ BMI-SDS	0.040	−5.095	0.040	0.086	−6.832	0.039
Δ WC (cm)	0.024	−0.500	0.111	0.061	−0.478	0.182
Δ HC (cm)	0.082	−1.154	0.003	0.108	−0.805	0.010
Δ fat (%)	0.009	−0.513	0.343	0.047	−0.328	0.568
Clinical variables						
Δ TAG (mg/dL)	0.041	−0.070	0.060	0.080	−0.055	0.188
Δ cholesterol (mg/dL)	0.077	−0.157	0.009	0.150	−0.194	0.004
Δ LDL (mg/dL)	0.059	−0.177	0.024	0.130	−0.211	0.015
Δ HDL (mg/dL)	0.004	−0.108	0.570	0.079	−0.264	0.197
Δ non-HDL (mg/dL)	0.075	−0.191	0.006	0.152	−0.221	0.005
Δ glucose (mg/dL)	0.005	−0.106	0.522	0.055	−0.099	0.566
Δ QUICKI index	0.059	126.443	0.032	0.071	145.393	0.020
Δ HOMA index	0.056	−1.437	0.037	0.116	−1.364	0.061
Δ insulin (mg/dL)	0.071	−0.421	0.017	0.121	−0.370	0.048
Δ SBP (mmHg)	0.134	−0.359	<0.001	0.196	−0.366	<0.001
Δ DBP (mmHg)	0.074	−0.295	0.006	0.104	−0.264	0.026
Diet quality						
Δ DQI-A (−33% to 100%)	0.084	0.340	0.003	0.118	0.353	0.006
Δ HLD-I (0 to 36 points)	0.238	1.600	<0.001	0.253	1.573	<0.001
Δ KIDMED (0 to 12 points)	0.324	2.865	<0.001	0.340	2.859	<0.001

BMI-SDS, standard deviation score for body mass index; WC, waist circumference; HC, hip circumference; TAG, triglycerides; LDL, low density cholesterol; HDL, high density cholesterol; non-HDL, total cholesterol minus HDL cholesterol; QUICKI index, quantitative insulin sensitivity check index; HOMA index, homeostasis model assessment; SBP, systolic blood pressure; DBP, diastolic blood pressure; DQI-A, diet quality index, adapted for adolescents; HLD-I, healthy diet index; KIDMED, Mediterranean diet quality index for children; UPF, ultra-processed food. Regression model was adjusted for change in energy intake and somatic maturity.

### 3.6 Associations between Life’s Essential 8 and ultra-processed food consumption

**Figure 2:**
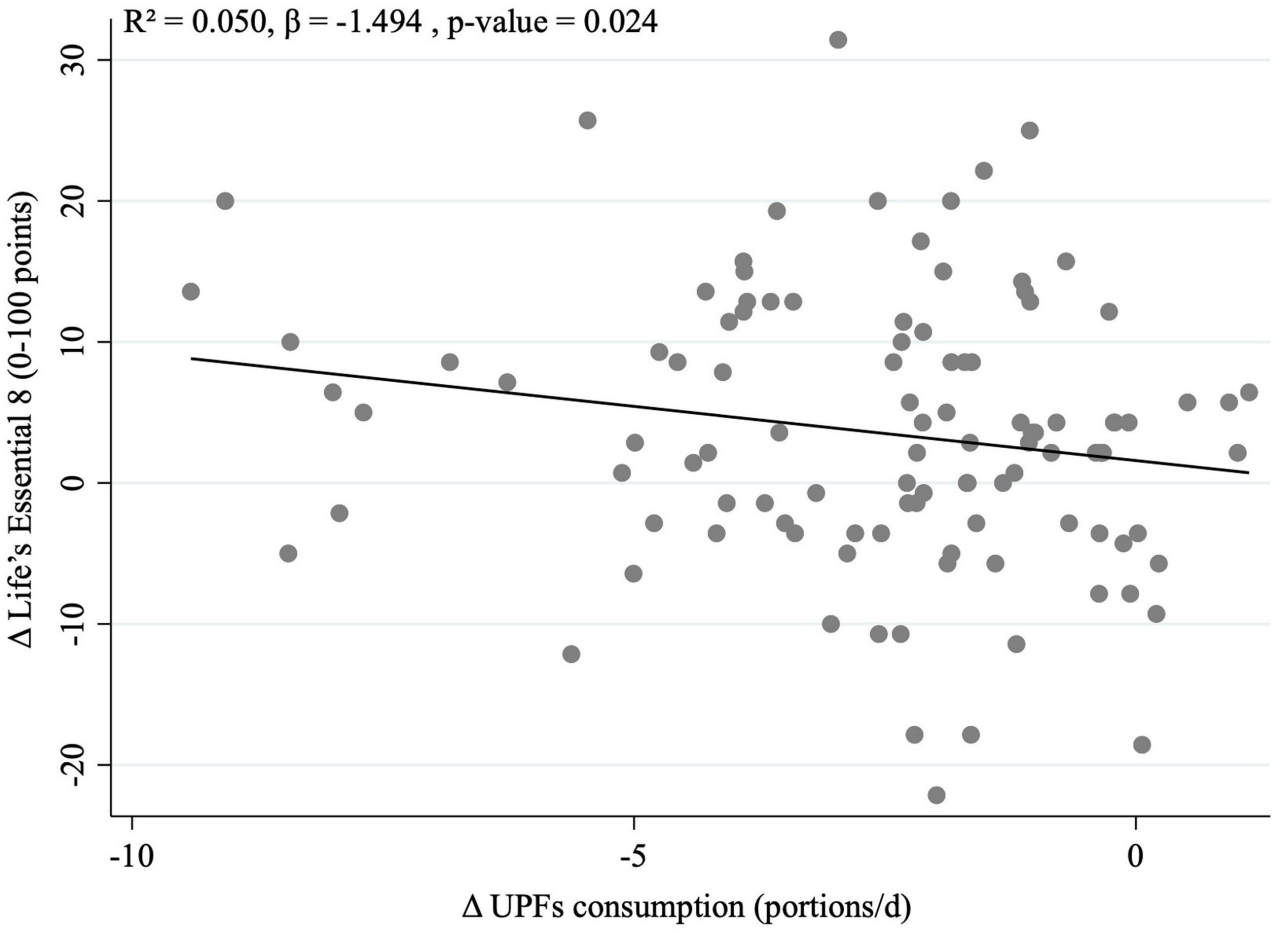
Association between changes in Life’s Essential 8 score and changes in ultra-processed food consumption in a pediatric population with adnominal obesity after the 8-week intervention. Regression model was adjusted for change in energy intake and somatic maturity.

Finally, a multivariable-adjusted model was used to evaluate the possible association between changes in UPF consumption and changes in LE8 (Figure 2). A significant negative association between changes in UPF consumption and changes in LE8 was found (*p* = 0.024) after adjusting for change in energy intake and somatic maturity. Notably, we observed that each decrease of one portion/day in UPF consumption was independently associated with an increase of 1.5 points in the LE8.

### 3.7 Mediation analysis

**Figure 3:**
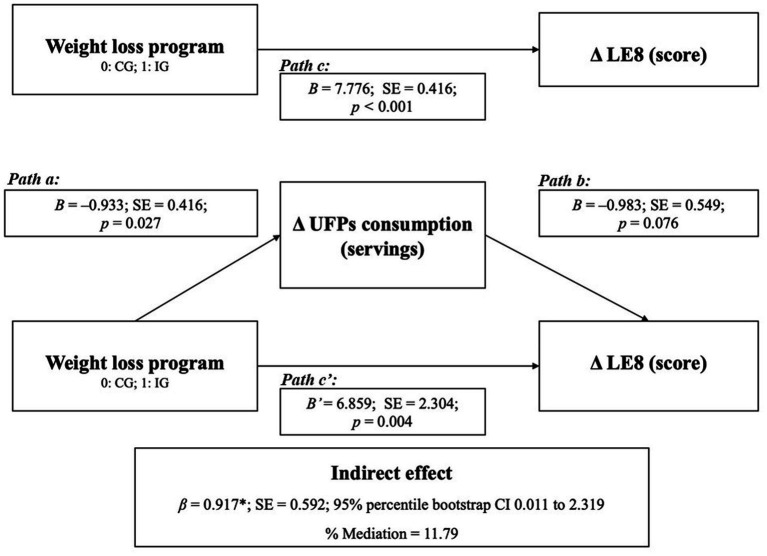
Mediation analysis (PROCESS Model 4) examining the indirect effect of changes in ultra-processed food consumption on the association between the weight loss program and changes in LE8 score. Path a represents the effect of the weight loss program on changes in UPFs consumption; path b represents the effect of changes in UPFs consumption on LE8 score; path c represents the total effect; and path c’ represents the direct effect. Indirect effects (ab) were estimated using 10,000 bootstrap samples. Adjusted for change in energy intake and somatic maturity. B, unstandardized beta coefficient; CI, confidence interval; LE8, Life’s Essential 8; SE, standard error; UPFs, ultra-processed foods.

Regarding our mediation analysis, we found that the weight loss program has a significant positive total effect on LE8 score (*β* = 7.776; *p* < 0.001; path c). The program also significantly reduced UFP consumption (*β* = −0.933; *p* = 0.027; path a). When including both the weight loss program and the change in UPF consumption in the regression model, the effect of UPF consumption on LE8 was not statistically significant (*β* = −0.983; *p* = 0.076; path b). The weight loss program continued to have a significant direct effect on LE8 (*β* = 6.859; *p* = 0.004; path c’), although this effect was attenuated by the reduction in UPF consumption. The indirect effect through UPF reduction was significant (*β* = 0.917; 95% percentile bootstrap CI 0.011 to 2.319), indicating that approximately 11.8% of the total effect of the weight loss program on LE8 was mediated by changes in UPF consumption (Figure 3).

## 4 Discussion

To our knowledge, this is the first study to explore the impact of a lifestyle intervention on CVH in children and adolescents with abdominal obesity using the AHA’s new LE8 metric. The study demonstrates that the improvement in LE8 associated with the program was partially mediated by reductions in UPF consumption.

In pediatric patients with abdominal obesity, the IGENOI lifestyle treatment significantly improved CVH, with the intervention group achieving a higher score in LE8. Prior to this study, no intervention had evaluated changes in CVH using AHA’s LE8 metrics. However, seven cohort studies have examined LE8 in children and adolescents, including three prospective studies (10–12) and four cross-sectional analyzes (13–16). Our study reported an overall CVH score of 63.6 at baseline and 67.5 at the end of intervention, which is lower than the scores reported in five of the seven cohort studies (ranging from 70–80 points) (10–13, 16). This lower score may be attributed to the presence of abdominal obesity in all participants. Two studies reported similar scores: Song et al. (15) reported a score of 63 points in children with adverse childhood experiences. Reported a score of 65.5 points, where low score was possibly due to the use of only three metrics: BMI, physical activity and diet Lloyd-Jones et al. (14). These three items had the lowest score across the research works (10–13, 15). Our study’s lowest scores were in the BMI category being 27.59 and 25.57, followed by the diet score being 48.54 and 37.00 at baseline in the intervention group and in the usual care group, respectively. These findings highlight the importance of lifestyle modification, especially diet quality, for preventing and treating obesity at this stage.

Additionally, our study emphasizes the clinical relevance of LE8 as a robust measure of children’s CVH. We found significant associations between changes in LE8 and various key health indicators and lifestyle behaviors, such as anthropometric measurements, lipid profiles, glucose metabolism markers, BP, and diet quality indexes. This aligns with findings by Sun et al., who demonstrated the validity of LE8 in assessing childhood CVH through its association with cardiovascular structural measurements (12).

The lifestyle intervention, which include a Mediterranean diet, improved CVH in pediatric patients with abdominal obesity. This effect was partially mediated by the reduction in UPF consumption. To our knowledge, no other study has evaluated the effect of a lifestyle intervention on CVH, or the mediating role of diet in this context. The IGENOI intervention successfully reduced UPF consumption among all participants, with a greater decrease observed in the intervention group, correlated with the improvement in CVH. Recent reviews on UPF consumption in children and adolescents have consistently indicated a positive association between UPF intake and various cardiometabolic comorbidities, such as dyslipidemia, BP, diabetes, and metabolic syndrome (17). To date, only one cross-sectional study has examined the association between UPF consumption and the AHA’s LE7 metrics, with Zhang et al. (18) demonstrating a graded inverse association between the UPF calorie percentage and CVH in a population of adolescents aged 12–19 years from the NHANHES study.

Our findings provide novel insights into the mediation role of UPF consumption in lifestyle interventions targeting pediatric obesity. Few randomized controlled trials have evaluated potential changes in UPF consumption (39–41). However, individualized plans and educational activities have shown promise in reducing UPF intake among children with obesity (39). School-based nutrition interventions have also been effective in improving dietary intake, specifically by increasing the consumption of unprocessed food and decreasing UPF consumption (40). Moreover, poor sleep quality in pediatric obesity has recently been associated with increased caloric intake, particularly from UPF (41). Despite these findings, the potential mediation of UPF consumption in lifestyle interventions has not been thoroughly investigated. Our results highlight the importance of modifying incorrect dietary habits through lifestyle interventions to improve cardiometabolic health in pediatric obesity.

Our study has several strengths, including (1) a longitudinal design allowing for paired comparisons of participants with baseline data, (2) the involvement of registered nutritionists in collecting dietary data through a validated FFQ, and (3) the use of LE8 in a pediatric population, with objective measurements of physical activity levels and sleep duration. Several limitations should be acknowledged. Participants varied in age and pubertal stage, which may have influenced lifestyle behaviors and cardiometabolic outcomes; to mitigate this, somatic maturity assessed by pediatricians using the Tanner scale was included as a covariate in all statistical models. A relevant limitation of this study is the use of modified versions of the HLD-I and LE8 scores, which have not undergone a formal psychometric re-validation. The modification of existing indices implies that these versions should be considered new operationalizations of the underlying constructs. In particular, the absence of a formal validation process, especially with regard to measurement properties and cross-cultural adaptation to the Spanish context, requires that the findings derived from these modified scores be interpreted with appropriate caution, particularly when comparing results across studies using different versions of the indices. Consequently, the application of these modified indices in the present study should be regarded as exploratory, as no formal assessment of their measurement models (e.g., formative model evaluation or structural equation–based validation) was conducted, and the study was not designed for instrument validation purposes. Nevertheless, both scores are showed consistent and empirically coherent associations with relevant clinical and anthropometric outcomes in our population, supporting their interpretability in this specific context. Furthermore, as previously reported Zheng et al. (37), overall cardiovascular health can be accurately estimated even when one component of the LE8 score is missing, supporting the interpretation of our modified LE8 as a meaningful measure of CVH in pediatric populations. Practical implications.

Targeted lifestyle interventions aimed at reducing UPF consumption and promoting healthy behaviors may represent a feasible and effective strategy to improve cardiometabolic health in children and adolescents with abdominal obesity. The observed improvements in anthropometric measures, lipid profile, insulin sensitivity, and overall CVH suggest potential clinical relevance of incorporating structured dietary guidance and lifestyle counseling into pediatric obesity management. Moreover, the application of the AHA LE8 metric in this context illustrates its potential usefulness as an exploratory tool for monitoring changes in CVH and lifestyle-related risk factors in both clinical practice and public health–oriented interventions, while acknowledging that its modified version has not undergone formal psychometric validation.

## 5 Conclusion

In conclusion, our study suggests that a short-term lifestyle intervention in a pediatric population with abdominal obesity was associated with improvements in anthropometric and clinical parameters, as well as a significant reduction in UPF consumption. Using the AHA LE8 metric, we showed that the intervention’s effect on cardiovascular health was partially mediated by changes in UPF intake. Additionally, changes in LE8 were associated with cardiometabolic indicators and lifestyle behaviors, providing exploratory evidence supporting the relevance of this metric for assessing CVH in pediatric populations. Although the intervention period was relatively short and the mediation effect modest, these findings directly address the study objectives and indicate that even brief lifestyle interventions may contribute to meaningful improvements in diet quality and CVH.

## Data Availability

The original contributions presented in the study are included in the article/Supplementary material, further inquiries can be directed to the corresponding authors.
