# Supplementary material for: Decreased ultra-processed food consumption as a mediator for lowering cardiovascular risk after a lifestyle program in pediatric obesity: a randomized clinical trial
Source: Front Nutr. 2026 Feb 11;13:1753345. doi: 10.3389/fnut.2026.1753345 (PMC12932238; doi:10.3389/fnut.2026.1753345)
Supplement: Supplementary file 1 [file Table_1.docx]

**SUPPLEMENTARY DATA**

**Supplementary Table 1.** **Description of the dietary plan applied in intervention subjects.**

| Total energy expenditure | Basal metabolism (Schofield, 1985) assuming a PA factor (1.3) |
| --- | --- |
| Calorie restriction according to BMI-SDS (Marqués *et al.*, 2012) | % of energy restriction of total daily energy expenditure |
| +1 SDS  +2 SDS  +3 SDS  ˃+4 SDS | -10%  -20%  -30%  -40% |
| Total daily energy distribution (Serra-Majem, 2011) | % of total energy |
| Breakfast  Morning snack  Lunch  Afternoon snack  Dinner | 20%  5-10%  30–35%  10–15%  20–25% |
| Well-balanced distribution of macronutrients (Serra-Majem, 2011) | % of total energy |
| Carbohydrates  Fat  Proteins | 55%  30%  15% |
| Energy-adjusted full-day menu based on Mediterranean Pattern (Willett *et al.*, 1995) |  |
| High consumption  Moderate consumption  Low consumption | Fruit, vegetables, whole grains, legumes, nuts, seeds and olive oil and minimally processed foods  Dairy products, fish and poultry  Red meat |

**References:**

Marqués M, Moleres A, Rendo-Urteaga T, et al. Design of the nutritional therapy for overweight and obese Spanish adolescents conducted by registered dieticians: the EVASYON study. Nutr Hosp. 2012;27(1):165-176. doi:10.1590/S0212-16112012000100020.

Schofield WN. Predicting basal metabolic rate, new standards and review of previous work. *Hum Nutr Clin Nutr*. 1985;39(1):5-41.

Serra-Majem L, Ribas L, Ngo J, et al. Food, youth and the Mediterranean diet in Spain. Development of KIDMED, Mediterranean Diet Quality Index in children and adolescents. *Public Health Nutr*. 2004;7(7):931-935. doi:10.1079/PHN2004556.

Willett WC, Sacks F, Trichopoulou A, Drescher G, Ferro-Luzzi A, Helsing E, Trichopoulos D. Mediterranean diet pyramid: a cultural model for healthy eating. *Am J Clin Nutr*. 1995;61(6):1402S-1406S. doi: 10.1093/ajcn/61.6.1402S.

**
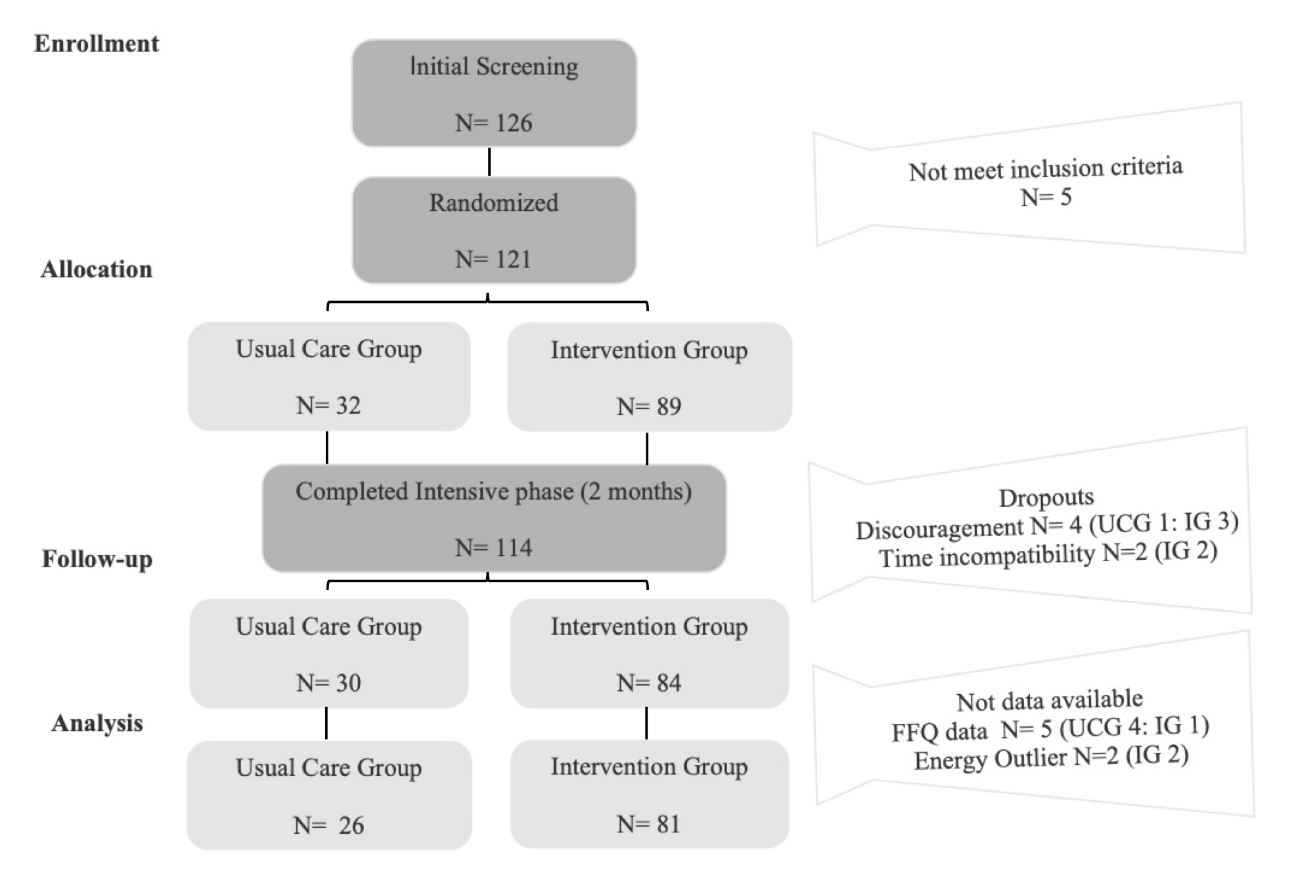
**

**Supplementary Figure 1.** **Flowchart of IGENOI study.** UGC, Usual Care Group; IG, Intervention Group; FFQ, Food Frequency Questionnaire
